# Dataset on the carbon dioxide, methane and nitrogen high-pressure sorption properties of South African bituminous coals

**DOI:** 10.1016/j.dib.2019.104248

**Published:** 2019-07-13

**Authors:** Gregory N. Okolo, Raymond C. Everson, Hein W.J.P. Neomagus, Richard Sakurovs, Mihaela Grigore, John R. Bunt

**Affiliations:** a Center of Excellence in Carbon Based Fuels, Unit for Energy and Technology Systems (UETS), School of Chemical and Minerals Engineering, North-West University, Potchefstroom Campus, Private Bag X6001, Potchefstroom, 2520, South Africa; b CSIRO Energy, 11 Julius Avenue, North Ryde NSW 2113, Australia

**Keywords:** CO_2_ storage in coal seam, Excess sorption isotherm, Maximum sorption capacity, Dubinin-radushkevich-henry law hybrid (DR-HH)

## Abstract

The dataset presented in this article supplements the result and information published in the report “The carbon dioxide, methane and nitrogen high-pressure sorption properties of South African bituminous coals” (Okolo et al., 2019). Four run of mine coal samples from selected underground coal mines from the Highveld, Witbank, and Tshipise-Pafuri coalfields of South Africa were used for the study. The CO_2_, CH_4_, and N_2_ sorption data were acquired from an in-house built high-pressure gravimetric sorption system (HPGSS) at the CSIRO Energy, North Ryde, Australia; at an isothermal temperature of 55 °C, in the pressure range: 0.1–16 MPa. The resulting excess sorption isotherm data were fitted to the modified Dubinin-Radushkevich isotherm model (M-DR) and a new Dubinin-Radushkevich – Henry law hybrid isotherm model (DR-HH). The dataset provided in this article, apart from being informative will be useful for comparison with available and future data and for testing other sorption isotherm models developed by other investigators in the area of CO_2_ storage in geological media, especially coal seams.

**Table:** Specifications table

Subject area	*Chemical Engineering, Energy, Environmental Science*
More specific subject area	*Carbon Capture, utilization and storage*
Type of data	*Table, graph, figure*
How data was acquired	*In-house built high-pressure gravimetric sorption system (HPGSS), Quantachrome Instruments Ultrapyc 1200e gas pycnometer.*
Data format	*Filtered-raw, systematically analyzed*
Experimental factors	*As-received Run of mine (ROM) coal samples were prepared to obtain representative* 1 mm *average particle size fractions that were used for both the density measurements and sorption experiments following the procedure detailed in our previous reports*[1], [2], [3]*. Sorption data were logged from the HPGSS at regular intervals. After data collection, non-equilibrium data were filtered off, and systematically analysed to get the adsorbate gases' sorption isotherm data in kg/t_coal_.*
Experimental features	*Sorption experiments were conducted on the samples at an isothermal temperature of 55 °C in the pressure range: 0.1 –* 16 MPa *[1]**.*
Data source location	*Potchefstroom, South Africa. North West University, Potchefstroom Campus.* *North Ryde, Sydney, NSW, Australia. CSIRO Energy*
Data accessibility	*Data are with this article*
Related research article	*The carbon dioxide, methane and nitrogen high-pressure sorption properties of South African bituminous coals**[1]**.*

**Table:** 

**Value of the data** • This research data gives insight into the sorption properties of typical South African bituminous coal. • The data provided can be used by other researcher as a benchmark for future work. • The dataset can be used for comparison with other available or determined data. • The sorption data presented can be tested with other empirical, available or developed isotherm model(s) for assessment and appraisal.

## 1 Data

**Fig. 1:**
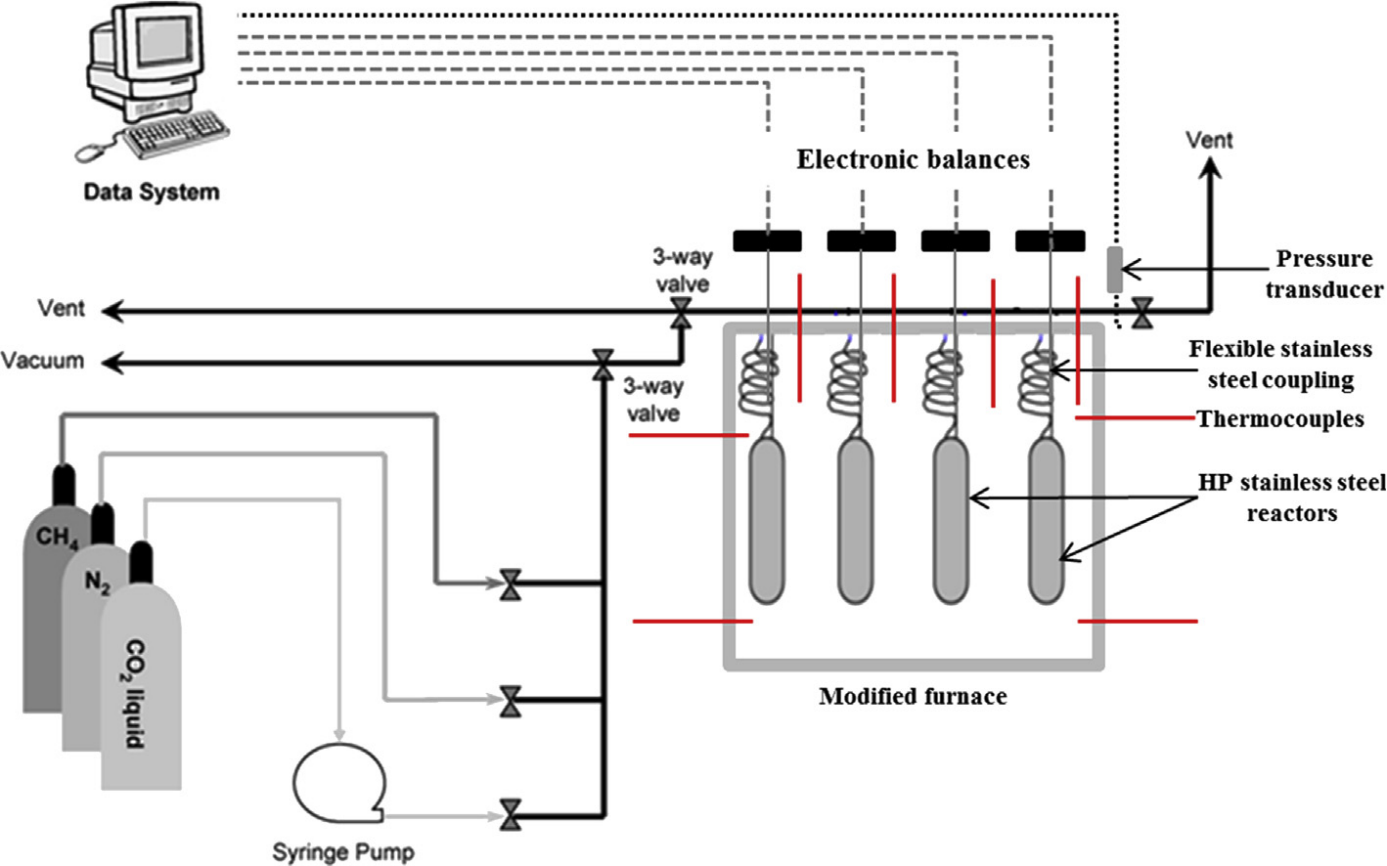
Schematic diagram of the high-pressure gravimetric sorption system (HPGSS) (Adapted from Day et al. [4]; *Not drawn to scale*).

**Fig. 2:**
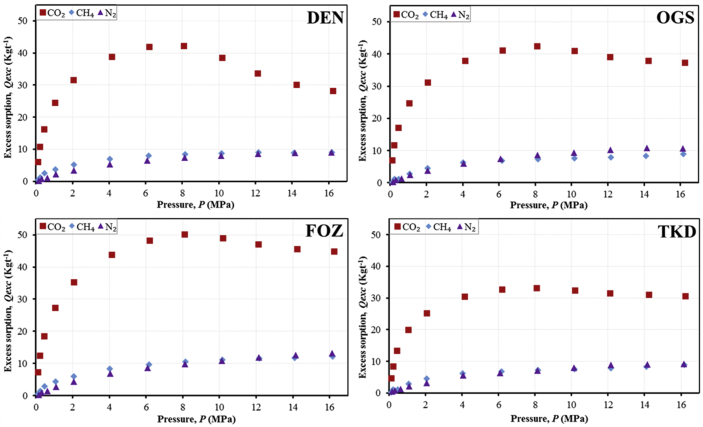
Comparison of the CO_2_, CH_4_, and N_2_ excess sorption isotherms for each sample.

**Fig. 3:**
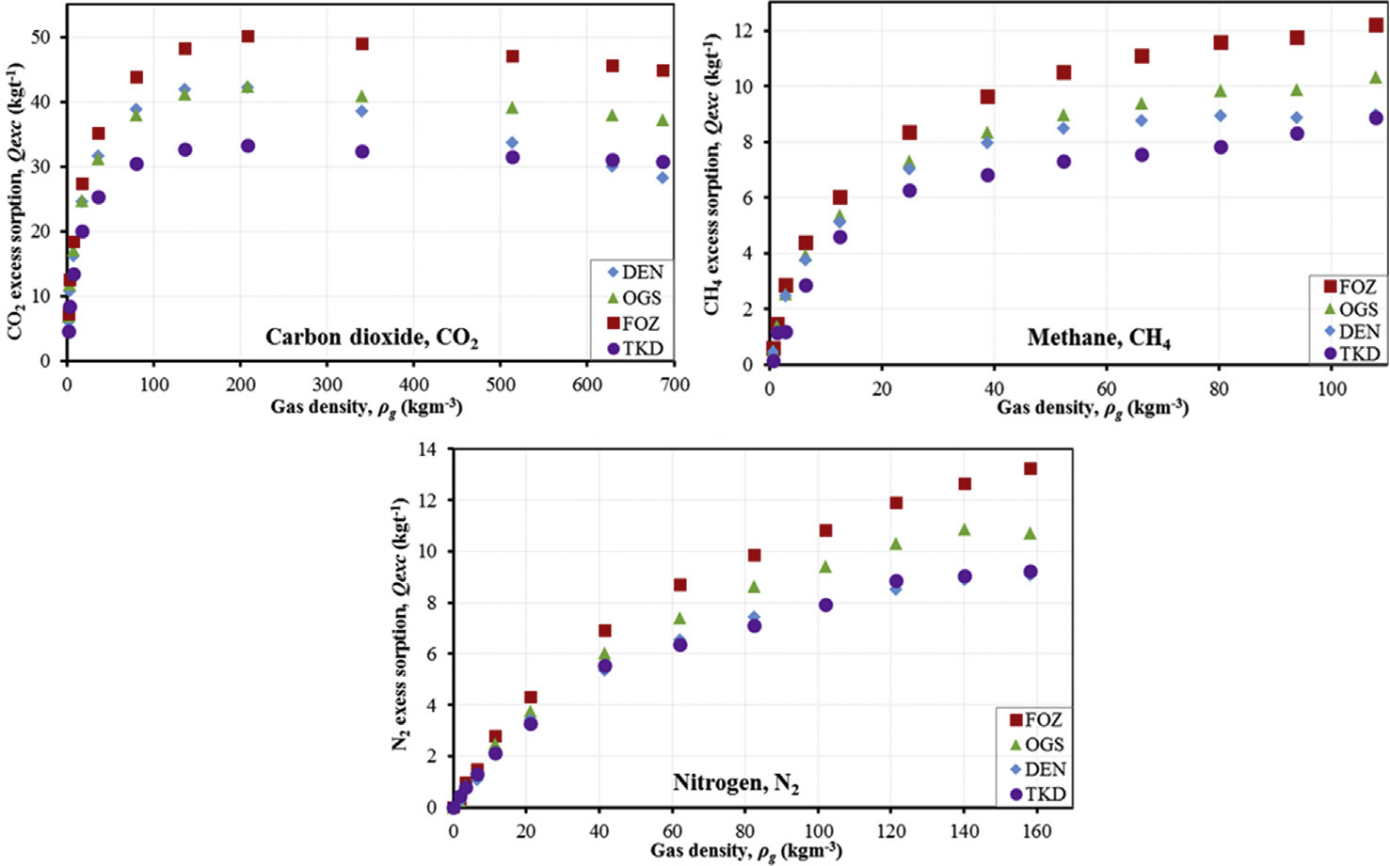
Comparison of the CO_2_, CH_4_, and N_2_ excess sorption isotherms of the samples with respect to the adsorbate gases.

**Fig. 4:**
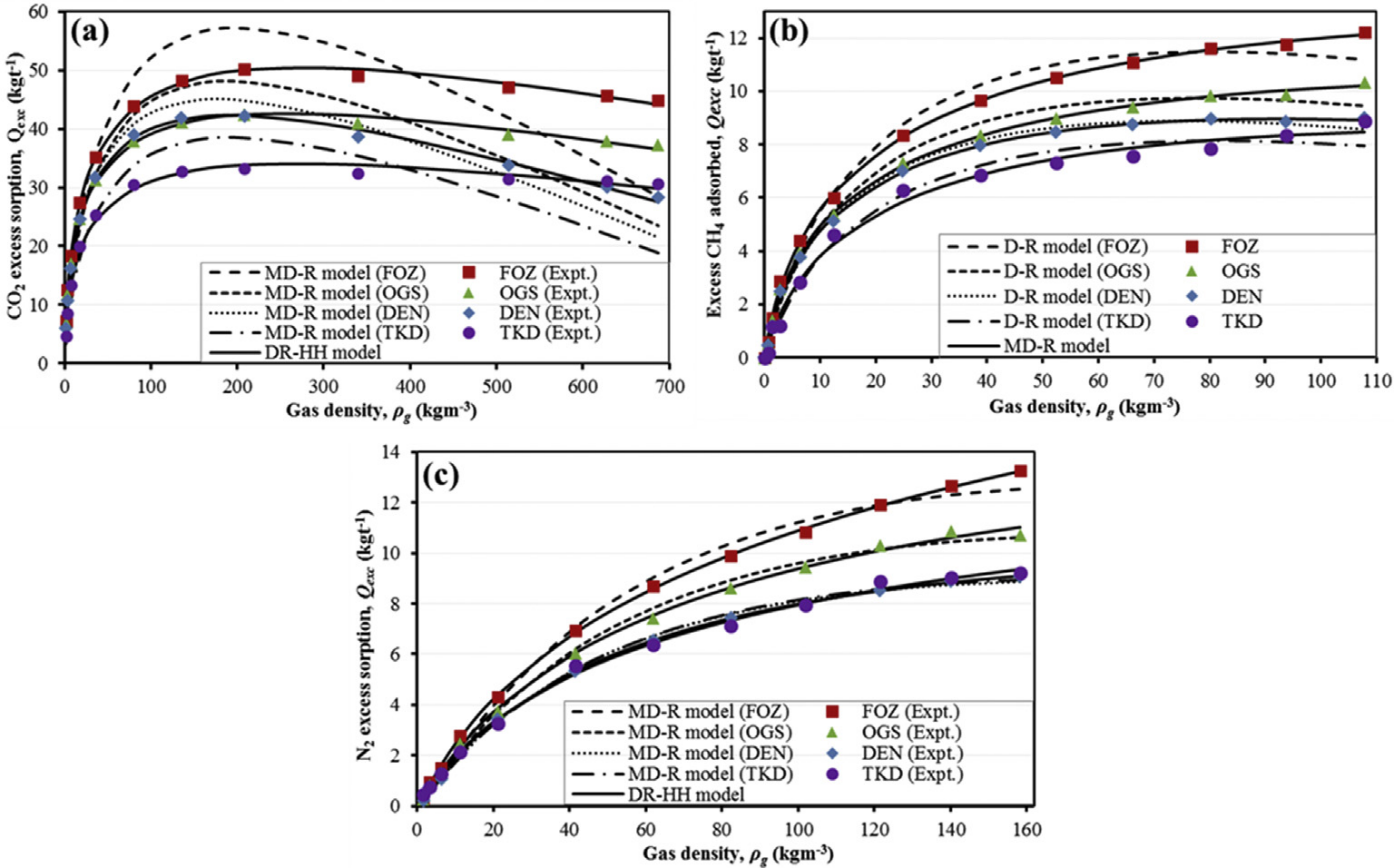
M-DR and DR-HH isotherm model fittings to the experimental (a) CO_2_, (b) CH_4_, and (c) N_2_ experimental excess sorption data of the coal samples.

The research dataset presented in this data report supplements the result and information published in the International Journal of Coal Geology [1], and consists of 5 tables, 1 figure, and 3 graphs (total 4 figures). Fig. 1 shows a not-drawn-to-scale schematic of the HPGSS adapted from Day et al. [4], while Table 1 presents the experimental factors, including sample mass and density. In Table 2, the systematically analysed data from the HPGSS for the 3 adsorbate gases (CO_2_, CH_4_, and N_2_) are presented. Fig. 2, Fig. 3 show the experimental sorption isotherms of the samples for the three gases, and further compares the resulting isotherms with regards to the 4 coals, as well the 3 adsorbate gases. In Table 3, Table 4, Table 5, the M-DR and DR-HH excess sorption isotherm model fittings data are provided for the 3 gases, while Fig. 4 depicts the graphical representation of the M-DR and DR-HH excess sorption isotherm fittings to the experimental excess sorption data of the coal samples for all three adsorbate gases (CO_2_, CH_4_, and N_2_) in 2–D rendering.

**Table 1:** Experimental factors.

Sample ID/parameters	DEN	OGS	FOZ	TKD
Sample weight (g)	225.29	229.04	220.48	225.45
Density (kgm^−3^)	1685.4	1574.6	1533.3	1487.6
Sample cell location	Cell #1	Cell #2	Cell #3	Cell #4
Isothermal temperature (°C)	55 °C

**Table 2:** CO_2_, CH_4_, and N_2_ excess sorption isotherm data of the coal samples.

Carbon dioxide (CO_2_) excess sorption, *Q_exc_* (kg t^−1^) (db)					
Pressure, *P* (MPa)	Gas density, *ρ_g_* (kg m^−3^)	DEN	OGS	FOZ	TKD
0.1052	1.7036	6.0806	6.9664	7.2911	4.6445
0.2200	3.5776	10.7999	11.7390	12.5281	8.4804
0.4391	7.1986	16.2837	17.1022	18.4398	13.3907
1.0537	17.6856	24.5218	24.7386	27.3530	20.0485
2.0561	35.9626	31.6399	31.2187	35.2323	25.2720
4.1348	79.8852	38.9013	37.9850	43.8449	30.4186
6.1843	135.4973	41.9272	41.1873	48.2612	32.6679
8.0911	207.9895	42.1776	42.4067	50.1199	33.2439
10.1733	339.8949	38.6201	40.9607	48.9972	32.4170
12.1105	513.7378	33.7485	39.1124	47.0691	31.4816
14.2367	628.0571	30.1006	37.9188	45.6193	31.0692
16.2377	687.2398	28.2556	37.2927	44.8803	30.6997

Methane (CH_4_) excess sorption, *Q_exc_* (kg t^−1^) (db)					
Pressure, *P* (MPa)	Gas density, *ρ_g_* (kg m^−3^)	DEN	OGS	FOZ	TKD
0.1132	0.6667	0.4626	0.5353	0.5931	0.1540
0.2315	1.3654	1.1887	1.4192	1.4724	1.1590
0.4750	2.8093	2.4788	2.5649	2.8562	1.1789
1.0787	6.4262	3.7639	3.9360	4.3866	2.8467
2.0661	12.4510	5.1266	5.3528	6.0100	4.6008
4.0324	24.8426	7.0223	7.3055	8.3525	6.2769
6.1552	38.7681	7.9628	8.3468	9.6534	6.8408
8.1611	52.3585	8.4773	8.9687	10.5230	7.3252
10.1568	66.1680	8.7613	9.4076	11.0862	7.5526
12.1650	80.1984	8.9539	9.8511	11.5986	7.8396
14.1102	93.7198	8.8566	9.8813	11.7484	8.3331
16.1754	107.8090	8.9885	10.3417	12.2172	8.8618
Nitrogen (N_2_) excess sorption, *Q_exc_* (kg t^−1^) (db)					

Pressure, *P* (MPa)	Gas density, *ρ_g_* (kg m^−3^)	DEN	OGS	FOZ	TKD
0.1684	1.7289	0.2238	0.2612	0.3178	0.4433
0.3188	3.2734	0.7251	0.8487	0.9541	0.7732
0.6249	6.4174	1.0927	1.3153	1.4904	1.2927
1.1028	11.3211	2.1631	2.4872	2.7902	2.1388
2.0654	21.1906	3.4196	3.7688	4.3234	3.2797
4.0553	41.5046	5.3570	6.0511	6.9245	5.5327
6.0860	62.0460	6.5543	7.4166	8.7065	6.3771
8.1248	82.3925	7.4455	8.6282	9.8850	7.1077
10.1292	102.0160	7.9476	9.4314	10.8222	7.9509
12.1536	121.4200	8.5399	10.3162	11.9149	8.8667
14.1483	140.0341	8.9066	10.8615	12.6648	9.0379
16.1510	158.2013	9.0753	10.7315	13.2479	9.2338

**Table 3:** M-DR and DR-HH excess sorption isotherm model fittings data for CO_2_ experimental excess sorption data.

Pressure, *P* (MPa)	Gas density, *ρ_g_* (kgm^−3^)	DEN (CO_2_)			OGS (CO_2_)		
		Experimental excess sorption, *Q_exc_* (kgt^−1^) (db)	M-DR fitting (kgt^−1^) (db)	DR-HH fitting (kgt^−1^) (db)	Experimental excess sorption, *Q_exc_* (kgt^−1^) (db)	M-DR fitting (kg t^−1^) (db)	DR-HH fitting (kgt^−1^) (db)
0.1052	1.7036	6.0806	5.5292	7.0842	6.9664	4.3963	8.0316
0.2200	3.5776	10.7999	9.4612	11.1356	11.7390	8.0676	12.0618
0.4391	7.1986	16.2837	14.7451	16.1866	17.1022	13.3247	16.8980
1.0537	17.6856	24.5218	23.7851	24.2453	24.7386	22.9005	24.3754
2.0561	35.9626	31.6399	32.0643	31.2489	31.2187	32.1680	30.7866
4.1348	79.8852	38.9013	40.7069	38.3924	37.9850	42.3714	37.5065
6.1843	135.4973	41.9272	44.3324	41.5152	41.1873	47.0264	40.8765
8.0911	207.9895	42.1776	44.7251	42.2269	42.4067	48.0223	42.4218
10.1733	339.8949	38.6201	40.6563	39.9134	40.9607	44.1092	42.2191
12.1105	513.7378	33.7485	31.7767	34.2606	39.1124	34.6763	39.7987
14.2367	628.0571	30.1006	25.0701	29.9087	37.9188	27.4088	37.6858
16.2377	687.2398	28.2556	21.4643	27.5584	37.2927	23.4813	36.5122
Pressure, *P* (MPa)	Gas density, *ρ_g_* (kgm^−3^)	FOZ (CO_2_)			TKD (CO_2_)		
		Experimental excess sorption, *Q_exc_* (kgt^−1^) (db)	M-DR fitting (kgt^−1^) (db)	DR-HH fitting (kgt^−1^) (db)	Experimental excess sorption, *Q_exc_* (kgt^−1^) (db)	M-DR fitting (kgt^−1^) (db)	DR-HH fitting (kgt^−1^) (db)

0.1052	1.7036	7.2911	3.9272	8.1898	4.6445	3.3059	6.2127
0.2200	3.5776	12.5281	7.7049	12.7049	8.4804	6.1577	9.3805
0.4391	7.1986	18.4398	13.4515	18.2864	13.3907	10.2965	13.2017
1.0537	17.6856	27.3530	24.5622	27.1749	20.0485	17.9364	19.1438
2.0561	35.9626	35.2323	35.8854	35.0037	25.2720	25.4165	24.2717
4.1348	79.8852	43.8449	48.9620	43.4322	30.4186	33.7418	29.6993
6.1843	135.4973	48.2612	55.3324	47.8210	32.6679	37.5997	32.4777
8.0911	207.9895	50.1199	57.1634	50.0044	33.2439	38.4953	33.8250
10.1733	339.8949	48.9972	53.0289	50.1941	32.4170	35.4366	33.8648
12.1105	513.7378	47.0691	41.9207	47.7231	31.4816	27.8929	32.1928
14.2367	628.0571	45.6193	33.1939	45.4365	31.0692	22.0558	30.6755
16.2377	687.2398	44.8803	28.4547	44.1512	30.6997	18.8979	29.8260

**Table 4:** M-DR and DR-HH excess sorption isotherm model fittings data for CH_4_ experimental excess sorption data.

Pressure, *P* (MPa)	Gas density, *ρ_g_* (kgm^−3^)	DEN (CH_4_)			OGS (CH_4_)		
		Experimental excess sorption, *Q_exc_* (kgt^−1^) (db)	M-DR fitting (kgt^−1^) (db)	DR-HH fitting (kgt^−1^) (db)	Experimental excess sorption, *Q_exc_* (kgt^−1^) (db)	M-DR fitting (kgt^−1^) (db)	DR-HH fitting (kgt^−1^) (db)
0.1132	0.6667	0.4626	0.5796	0.6997	0.5353	0.5171	0.8068
0.2315	1.3654	1.1887	1.1364	1.2883	1.4192	1.0622	1.4314
0.4750	2.8093	2.4788	2.0576	2.2097	2.5649	2.0044	2.3795
1.0787	6.4262	3.7639	3.6542	3.7324	3.9360	3.7064	3.9134
2.0661	12.4510	5.1266	5.3041	5.2609	5.3528	5.5266	5.4489
4.0324	24.8426	7.0223	7.1433	6.9632	7.3055	7.6163	7.2103
6.1552	38.7681	7.9628	8.1640	7.9513	8.3468	8.8102	8.3191
8.1611	52.3585	8.4773	8.6559	8.4835	8.9687	9.4060	9.0021
10.1568	66.1680	8.7613	8.8659	8.7802	9.4076	9.6796	9.4731
12.1650	80.1984	8.9539	8.8884	8.9229	9.8511	9.7375	9.8075
14.1102	93.7198	8.8566	8.7857	8.9576	9.8813	9.6488	10.0370
16.1754	107.8090	8.9885	8.5857	8.9175	10.3417	9.4481	10.2075
Pressure, *P* (MPa)	Gas density, *ρ_g_* (kgm^−3^)	FOZ (CH_4_)			TKD (CH_4_)		
		Experimental excess sorption, *Q_exc_* (kgt^−1^) (db)	M-DR fitting (kgt^−1^) (db)	DR-HH fitting (kgt^−1^) (db)	Experimental excess sorption, *Q_exc_* (kgt^−1^) (db)	M-DR fitting (kgt^−1^) (db)	DR-HH fitting (kgt^−1^) (db)
0.1132	0.6667	0.5931	0.4990	0.8403	0.1540	0.3195	0.4817
0.2315	1.3654	1.4724	1.0731	1.5271	1.1590	0.7027	0.9275
0.4750	2.8093	2.8562	2.1086	2.5945	1.1789	1.4084	1.6577
1.0787	6.4262	4.3866	4.0567	4.3615	2.8467	2.7627	2.9240
2.0661	12.4510	6.0100	6.2109	6.1676	4.6008	4.2846	4.2595
4.0324	24.8426	8.3525	8.7547	8.2830	6.2769	6.1065	5.8426
6.1552	38.7681	9.6534	10.2477	9.6456	6.8408	7.1894	6.8499
8.1611	52.3585	10.5230	11.0153	10.5049	7.3252	7.7536	7.4645
10.1568	66.1680	11.0862	11.3883	11.1135	7.5526	8.0343	7.8785
12.1650	80.1984	11.5986	11.4949	11.5601	7.8396	8.1228	8.1609
14.1102	93.7198	11.7484	11.4180	11.8797	8.3331	8.0781	8.3433
16.1754	107.8090	12.2172	11.2024	12.1304	8.8618	7.9331	8.4662

**Table 5:** M-DR and DR-HH excess sorption isotherm model fittings data for N_2_ experimental excess sorption data.

Pressure, *P* (MPa)	Gas density, *ρ_g_* (kgm^−3^)	DEN (N_2_)			OGS (N_2_)		
		Experimental excess sorption, *Q_exc_* (kgt^−1^) (db)	M-DR fitting (kgt^−1^) (db)	DR-HH fitting (kgt^−1^) (db)	Experimental excess sorption, *Q_exc_* (kgt^−1^) (db)	M-DR fitting (kgt^−1^) (db)	DR-HH fitting (kgt^−1^) (db)
0.1684	1.7289	0.2238	0.2149	0.2896	0.2612	0.1969	0.3256
0.3188	3.2734	0.7251	0.5002	0.6138	0.8487	0.4855	0.6871
0.6249	6.4174	1.0927	1.0986	1.2407	1.3153	1.1248	1.3873
1.1028	11.3211	2.1631	1.9592	2.0894	2.4872	2.0869	2.3401
2.0654	21.1906	3.4196	3.3810	3.4330	3.7688	3.7403	3.8647
4.0553	41.5046	5.3570	5.4068	5.3128	6.0511	6.1867	6.0475
6.0860	62.0460	6.5543	6.7219	6.5608	7.4166	7.8217	7.5508
8.1248	82.3925	7.4455	7.5895	7.4318	8.6282	8.9228	8.6460
10.1292	102.0160	7.9476	8.1528	8.0495	9.4314	9.6518	9.4625
12.1536	121.4200	8.5399	8.5209	8.5098	10.3162	10.1394	10.1077
14.1483	140.0341	8.9066	8.7414	8.8472	10.8615	10.4418	10.6142
16.1510	158.2013	9.0753	8.8588	9.1005	10.7315	10.6143	11.0267

Pressure, *P* (MPa)	Gas density, *ρ_g_* (kgm^−3^)	DEN (N_2_)			OGS (N_2_)		
		Experimental excess sorption, *Q_exc_* (kgt^−1^) (db)	M-DR fitting (kgt^−1^) (db)	DR-HH fitting (kgt^−1^) (db)	Experimental excess sorption, *Q_exc_* (kgt^−1^) (db)	M-DR fitting (kgt^−1^) (db)	DR-HH fitting (kgt^−1^) (db)
0.1684	1.7289	0.3178	0.1919	0.4116	0.4433	0.2029	0.3491
0.3188	3.2734	0.9541	0.4930	0.8366	0.7732	0.4792	0.6963
0.6249	6.4174	1.4904	1.1872	1.6352	1.2927	1.0663	1.3327
1.1028	11.3211	2.7902	2.2667	2.7010	2.1388	1.9204	2.1613
2.0654	21.1906	4.3234	4.1751	4.3939	3.2797	3.3452	3.4417
4.0553	41.5046	6.9245	7.0762	6.8485	5.5327	5.3944	5.2285
6.0860	62.0460	8.7065	9.0554	8.6017	6.3771	6.7344	6.4497
8.1248	82.3925	9.8850	10.4074	9.9407	7.1077	7.6230	7.3447
10.1292	102.0160	10.8222	11.3142	10.9931	7.9509	8.2028	8.0204
12.1536	121.4200	11.9149	11.9299	11.8735	8.8667	8.5841	8.5636
14.1483	140.0341	12.6648	12.3199	12.6078	9.0379	8.8146	8.9989
16.1510	158.2013	13.2479	12.5508	13.2444	9.2338	8.9397	9.3618

## 2 Experimental design, materials, and methods

The HPGSS and the experimental procedure has been previously described in details [1], [2], [4], [5], [6]. Briefly, the prepared samples were dried and degassed in a vacuum oven at 60 °C for 2 weeks prior to the sorption experiments. After this, the samples were weighed and loaded into the sample cells, and placed in the isothermal oven environment maintained at the experimental temperature of 55 °C. Further degassing was continued on the samples in the sample cells in the oven at < 0.5 mbar for another 24 hr. The sorption experiments were started on the samples by a stepwise pressure increment from 0.1 MPa to 16 MPa. It should be noted the density of the samples were measured on a Quantachrome Instruments Ultrapyc 1200e gas pycnometer before drying and degassing. The sorption experiments on the samples were conducted in the order: firstly, CO_2_, then CH_4_, and lastly, N_2_. After each adsorbate gas exposure to the samples, the samples were degassed for a minimum of 48 hrs before the next gas is sorbed onto the sample. The HPGSS can hold four sample cells simultaneously, thus, gas sorption on all four samples were done at the same time.

Data logging from the HPGSS is automated with the aid of data logging hardware and software coupled to the system. Data logged from the facility include, mass gain, real time, pressure, and temperature. The resulting raw data was filtered to remove data acquired at non-equilibrium state. Only equilibrium data at constant mass over a long time (usually ≥ 8 hr) were collected and analysed. The excess sorbed amount was calculated using Equation (1) [1], [2], [7]:

(1) $$Q_{exc}=M_{mea}-\left(V_{cell}-V_{sample}\right)\rho_{g}$$

(2) $$Q_{exc}=Q_{0}\left(1-\frac{\rho_{g}}{\rho_{a}}\right)\exp\left[-D{\left(\ln\frac{\rho_{a}}{\rho_{g}}\right)}^{2}\right]$$

(3) $$Q_{exc}=Q_{0}\left(1-\frac{\rho_{g}}{\rho_{a}}\right)\exp\left[-D{\left(\ln\frac{\rho_{a}}{\rho_{g}}\right)}^{2}\right]+k\rho_{g}$$

Where, *Q*_*exc*_, is the excess (Gibbs’) sorption (kg); *M*_*mea*_, is the measured mass of adsorbate at a given pressure (kg); *V*_*cell*_, is the volume of sample cell (m^3^); *V*_*sample*_, is the volume of sample (m^3^); *Q*_*0*_, is the maximum sorption capacity by weight (kg/t_coal_); $\rho_{a}$, is the adsorbed phase density (kgm^-^^3^); $\rho_{g}$, is the adsorbate gas density (kgm^-^^3^); *D*, is the affinity constant (−); *k*, is the proportionality constant (ml/g).

The maximum sorption capacities of the samples for the 3 gases were determined by fitting the experimental excess sorption data to the M-DR (Equation (2)) and the DR-HH (Equation (3)) excess sorption isotherm models [1], [2], [7]. Numerical analysis and model fitting was accomplished using the Visual Basic for Application (VBA) macros that were scripted and executed in Microsoft™ Excel 2013.
